# Ethnobotanical Survey, Preliminary Physico-Chemical and Phytochemical Screening of *Salvia argentea* (L.) Used by Herbalists of the Saïda Province in Algeria

**DOI:** 10.3390/plants6040059

**Published:** 2017-12-05

**Authors:** Yasmina Benabdesslem, Kadda Hachem, Khaled Kahloula, Miloud Slimani

**Affiliations:** 1 Laboratoire de Biotoxicologie, Pharmacognosie et Valorisation Biologique des Plantes (LBPVBP), Département de Biologie, Faculté des Sciences, Université Dr. Tahar Moulay de Saida, BP 138 cité ENNASR, Saida 20000, Algeria; benabdesslem.yasmina@univ-saida.dz (Y.B.); khaled.kahloula@univ-saida.dz (K.K.); miloud.slimani@univ-saida.dz (M.S.); 2 Laboratoire des Productions, Valorisations Végétales et Microbiennes (LP2VM), Département de Biotechnologies Végétales, Université des Sciences et de la Technologie d’Oran Mohamed Boudiaf, B.P. 1505, El-Mn’aour, Oran 31000, Algeria

**Keywords:** *Salvia argentea* (L.), ethnobotanical servey, Saïda province, leaf powder, physico-chemical, phytochemical screening

## Abstract

An ethnobotanical study was carried out in the Saïda region among herbalists to evaluate the use of *Salvia argentea* (L.), a plant species native from North Africa belonging to the Lamiaceae family. Forty-two herbalists were interviewed individually, aged between 30 and 70 years, all males, 52.38% of them having received a secondary education level and having performing their duties for more than a decade. This study showed that *Salvia argentea* is used specifically in the treatment of diseases of the respiratory system. The leaves are the most commonly used part, usually in the form of powder and exclusively administered orally. The preliminary results of the physicochemical characterization and the phytochemical screening of the powdered leaves of *Salvia argentea* attest to their safety and confer them a guarantee of phytotherapeutic quality.

## 1. Introduction

The Algerian flora in general and the region of Saïda in particular, benefit from an important reserve of plants with aromatic and medicinal characteristics. Thus, medicinal plants occupy an important place in the Algerian pharmacopoeia. Even today, they play a decisive role in the treatment of certain pathologies. Despite being one of the most impressive reserves of plants throughout the world, only 10% have been studied for their pharmacological properties [1].

The region of Saïda, by its geographical location, offers a rich and diverse vegetation. Many aromatic and medicinal plants grow there spontaneously. Interest in these plants has grown steadily in recent years. 

Among these numerous medicinal plants, our study focused on *Salvia argentea* belonging to the Lamiaceae family which is a plant species originating to the Mediterranean region, in northwest Africa (northern Algeria, Morocco, and Tunisia), southern Europe (Spain, Portugal, South Italy, Sicily, Malta, Albania, Bulgaria, Slovenia, Croatia, Bosnia, Kosovo, Montenegro, Serbia, Macedonia, and Greece) and Western Asia (Turkey) [2]. This family is known for its richness in numerous chemical substances capable of demonstrating various remarkable pharmacological activities [3]. Among these substances, we mention the essential oils which mainly consist of oxygenated sesquiterpenes [2,4] and which are endowed with important biological properties, such as antimicrobial [5,6] and antioxidant activities [7].

The leaves of *Salvia argentea*, also commonly known as “Ferrache en neda”, are heavily covered with a silvery down, hence its name; the leaves are soft. This plant possesses sticky stems and inflorescences. Sterile upper verticillasters consist only of bracts. White-pink flowers possess corollas three times longer than the calyx [8]. This plant differs from *Salvia patula*, by its leaves which are not heart-shaped at the base; by the upper lip of the calyx with less unequal and more distant teeth; and by its connective, more strongly toothed to the point where it widens [9]. The roots of *Salvia argentea* are thick and tuberous, which makes them resistant to heat and drought, but sensitive to humidity during winter [10].

We carried out an ethno botanical survey with herbalists working with medicinal plants. The results of this survey will allow us to identify the potential roles of *Salvia argentea* in the traditional pharmacopoeia and its effects in prophylaxis. The survey was followed by a physico-chemical and phytochemical study to detect the presence of groups of chemical families in a drug preparation based on *Salvia argentea*.

## 2. Results and Discussion

### 2.1. Ethno Botanical Survey

The ethno botanical and ethno-pharmacological investigation carried out as part of this work aimed to promote the expertise of herbalists in the Saïda region and to seek out their knowledge and know-how with medicinal plants, particularly *Salvia argentea*.

#### 2.1.1. Frequency of Use of *Salvia argentea* According to Herbalist Profile

**Figure 1:**
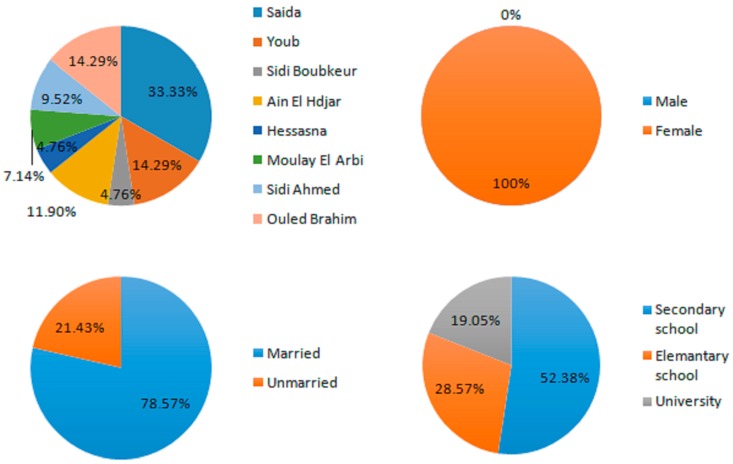
Information on surveyed herbalists.

This practice remains very important in the Saïda zone as evidenced by the number of herbalists surveyed (42), aged between 30 and 70 years, who practice their trade either in town or in the countryside. Herbalists are male (100%), most of them are married (78.57%) and a majority had received a secondary level education (52.38%) (Figure 1). All herbalists have been working for at least a decade, which sheds light on the accumulated experience and originality of knowledge about the use of *Salvia argentea*. They all have expressed the wish to follow continuous training either nationally or internationally and to develop collaboration with modern medicine through their participation in congresses and seminars.

#### 2.1.2. Type of Collectors of *Salvia argentea*

**Figure 2:**
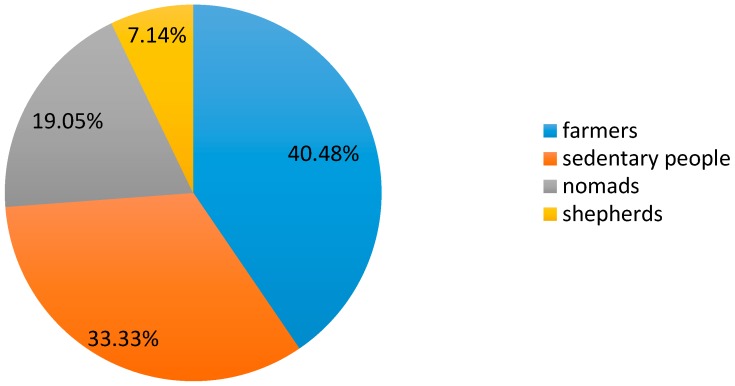
Type of *Salvia argentea* collectors.

Interviewed herbalists use several types of collectors (Figure 2): farmers (40.48%), sedentary people (33.33%), nomads (19.05%) and shepherds (7.14%).

#### 2.1.3. Use of *Salvia argentea* and Diseases Treated

All of the herbalists revealed that *Salvia argentea* is used in traditional medicine for therapeutic use, for the treatment of respiratory diseases. In the past, *Salvia argentea* leaves have been used against wounds, probably as a hemostatic [11], but no scientific validation has been reported so far.

#### 2.1.4. Opinion on the Efficacy of *Salvia argentea* in the Treatment of Respiratory Diseases

**Figure 3:**
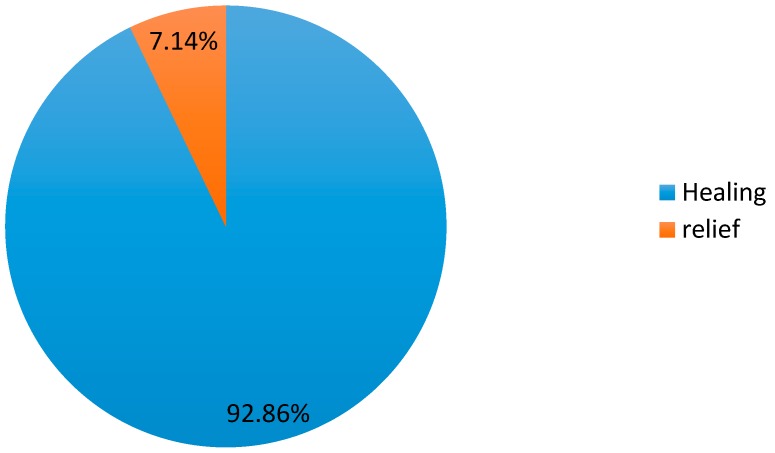
The opinion of herbalists on the efficacy of *Salvia argentea*.

The opinion of herbalists on the efficacy of *Salvia argentea* against respiratory diseases shows that 92.86% think that the traditional uses of this plant lead to a cure, while 7.14% say that the use of this plant has a relief effect only (Figure 3).

#### 2.1.5. The Part Used

**Figure 4:**
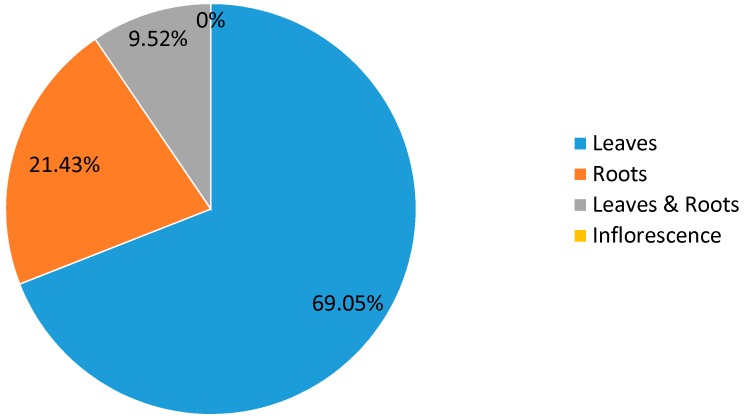
The used part of *Salvia argentea*.

The survey revealed that leaves are most commonly used for respiratory disease treatment with 69.05% (Figure 4), followed by roots (21.43%), and the whole plant (9.52%); however, there was no mention of any use for the inflorescences. This result is close to that established by Rhattas et al. who indicated that mainly the leaves of medicinal plants were used with a percentage of 71.75% [12]. This can be explained by the fact that leaves can be quickly harvested and that they are easy to use [13]; in addition, leaves are the main place of photosynthesis and the site of storage of many bioactive substances responsible for various biological properties [14]. The use of leaves is harmless for the regeneration of the plants and ensures the preservation of floristic richness [15]. Indeed, there is a clear relationship between the part of the plant which is exploited and the consequences of this exploitation on the persistence of this plant species [16].

#### 2.1.6. Method of Preparation and Administration

**Figure 5:**
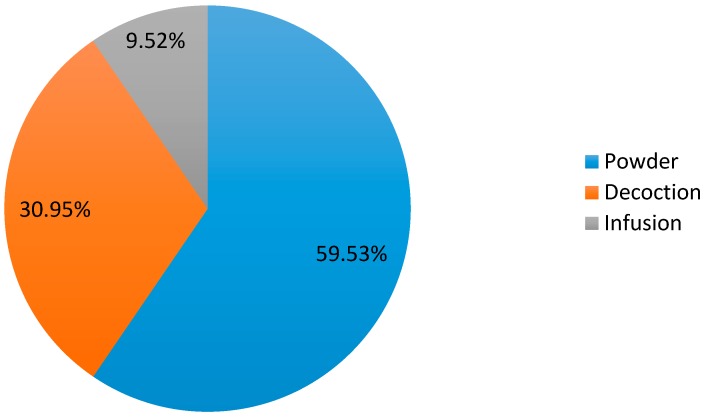
Method of preparation.

In Saïda, herbalists advocate several ways of preparing *Salvia argentea* for the treatment of respiratory diseases. Powder preparation is the most frequent mode (59.53%), followed by decoction (30.95%) and infusion (9.52%) (Figure 5). All the herbalists (100%) interviewed confirmed that the administration is exclusively oral. The best use of a plant would be that which preserves all its properties while allowing the extraction and assimilation of active compounds [17]. In addition, medicinal plants have side effects when they are incorrectly used by patients [12]. As a result, soft medicine must be practiced with care [18].

### 2.2. Physicochemical Characterization of the Leaf Powder of Salvia argentea

The results related to the physicochemical analyses of the leaf powder of *Salvia argentea* are reported in Table 1.

The values obtained for the water content are 12.89% on average. This low content assures that the powder of leaves of *Salvia argentea* can be preserved for a long time without great risk of alterations due to microbial contamination [19].

Total ash is the residue of mineral compounds remaining after incineration of a sample containing organic substances of animal, plant or synthetic origin. Total ash content represents about 17.61% on average of the dry mass. These values are comparable to those found with *Nasturtium officinale* (14.9–17.2%) and *Spinacia oleracea* (18.0–19.1%) [20].

The mean value obtained for the pH is 8.05. This value can be explained by the chlorophyll content of the leaves of *Salvia argentea*, which tends to confer basicity. The values of titratable acidity in term of lactic acid are also correlated with the pH value determined. Their average value is 0.74%. The same trend was observed by Houndji et al. in the leaf powder of *Moringa oleifera* (Lam.) [21].

### 2.3. Phytochemical Screening

The results of the phytochemical screening are presented in Table 2. They are classified according to various observation criteria, among others: very positive reaction (++++); positive reaction (+++); moderately positive reaction (++); doubtful reaction (+); and negative reaction (−).

The phytochemical screening carried out on the powder of the leaves of *Salvia argentea* shows the presence of chemical groups which possess interesting biological activities. These include alkaloids, anthocyanin flavonoids, saponins, coumarins, sterols and triterpenes, tannins (gallic and catechic acids) and reducing sugars. The complete absence of cyanogenetic derivatives greatly reduces the toxicological risks associated with the use of *Salvia argentea*.

The presence of potentially active chemical groups such as polyphenolic substances such as tannins in their two forms and anthocyanins in the powder of the studied leaves could justify the traditional indications of this plant by herbalists (survey) in traditional medicine, particularly for their pharmacological properties in the treatment of respiratory diseases [22,23]. Similarly, alkaloids and saponins have been recently reported to be helpful in fighting common pathogenic strains [24]. This plant is therefore a material of choice to enrich the conventional medicine with its interesting biological activities. 

## 3. Materials and Methods

### 3.1. Biological Material

**Figure 6:**
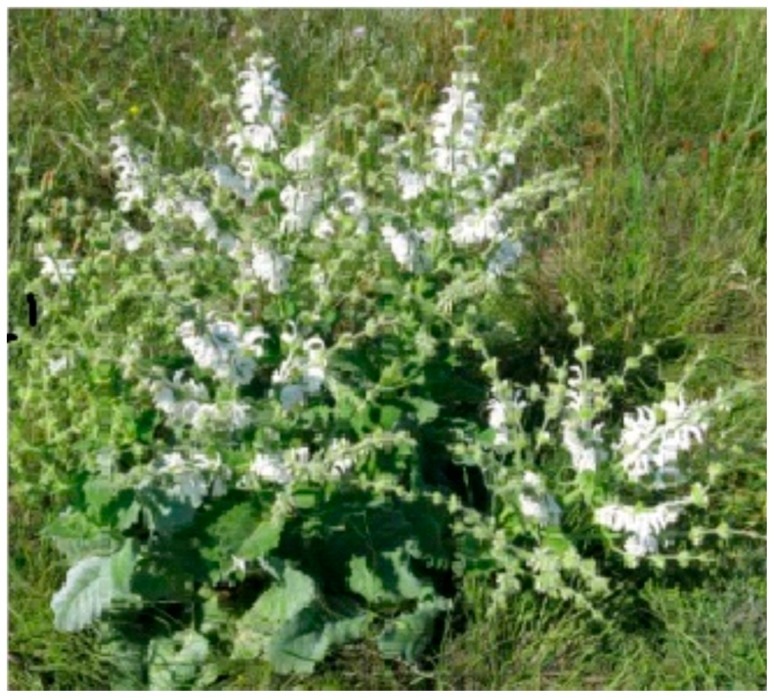
*Salvia argentea* at the full bloom stage.

The *Salvia argentea* samples used in the characterization section were harvested in the Saïda region in 2016, specifically in the Youb region. Plant harvesting was done at the full bloom stage (Figure 6). The identification of the plant was made by Prof. Hasnaoui O., botanist in the Department of Biology of the University of Saïda. A specimen of *Salvia argentea* is deposited in the herbarium of the department of biology of the university.

### 3.2. Ethnobotanical Investigation

#### 3.2.1. Study Area

**Figure 7:**
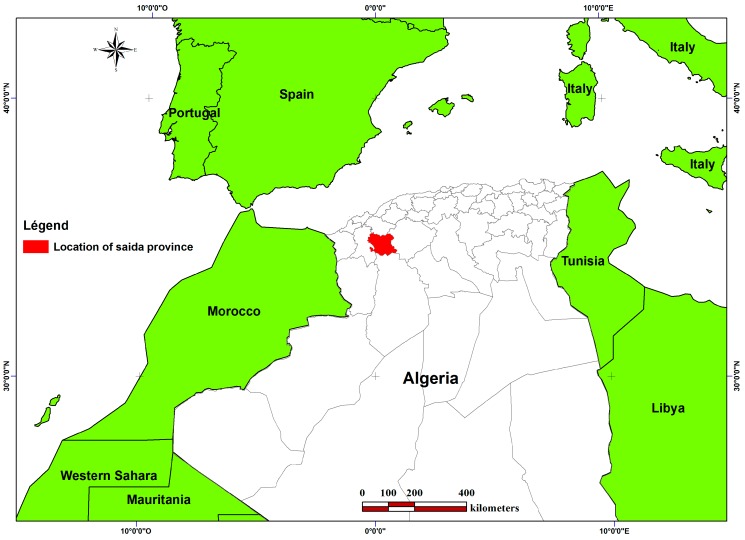
Situation map of Saïda Province.

Saïda Province, nicknamed the city of waters because of its numerous springs, is located in the Northwest part of Algeria (34°40′0″ N, 0°19′60″ E). With a population of 350.765, Saïda covers an area of 5536.73 km^2^ [25]. It is bordered to the north by the Mascara Province, to the south by the El Bayadh Province, to the west by the Sidi-Bel-Abbes Province and to the east by the Tiaret Province (Figure 7). This position gives it a role of relay between the steppe provinces in the south and the Tell provinces in the north; it allows the extension of the biodiversity of the plant species in this province. It contains 16 communes distributed at the level of 6 districts.

#### 3.2.2. Methods of Study

**Figure 8:**
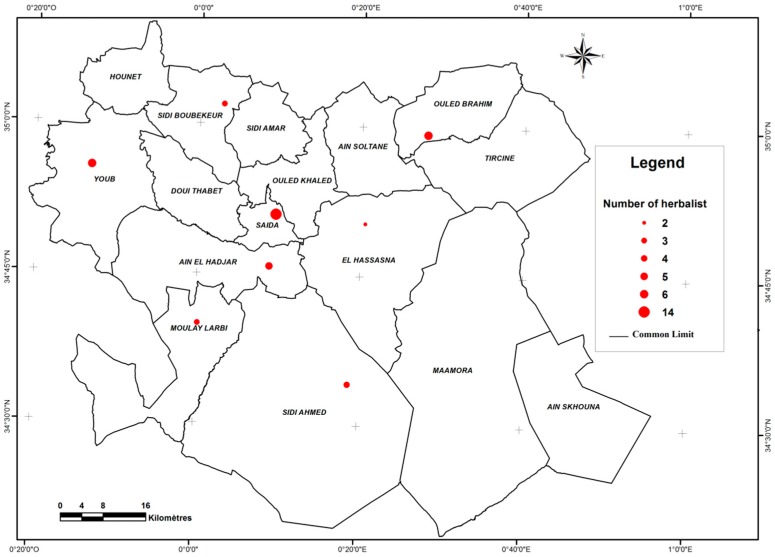
Distribution of survey points in the Saïda Province.

The ethnobotanical study was carried out by submitting a questionnaire to 42 herbalists, through 8 communes of the Saïda Province, 14 of them in Saïda, 6 in Youb, 2 in Sidi Boubkeur, 5 in Ain El Hdjar, 2 in Hessasna, 3 in Moulay El Arbi, 4 in Sidi Ahmed and 6 in Ouled Brahim (Figure 8). This disparity reflects the various population densities.

The survey questionnaire form (Appendix A) is divided into 8 parts to evaluate the knowledge of *Salvia argentea* in this area, the use, the prescription and preparation method recommended by each herbalist. All herbalists interviewed were informed about the purpose of this study. The raw data entered on the questionnaire forms were transferred to a database and processed by the Systematic Package for Social Sciences software (SPSS), version 10.

### 3.3. Characterization of Salvia argentea Leaf Powder

At the end of the ethnobotanical study, a preliminary characterization of some physico-chemical parameters (Humidity, ash, pH and titratable acidity) and phytochemical screening considered as basic analyses were carried out on the powder of the leaves of *Salvia argentea*. 

#### 3.3.1. Physico-Chemical Analyses

The humidity content of the previously dried and weighed leaf powder was determined by mass difference before and after desiccation in an oven at +103 °C until a constant mass was obtained [26]. The ash rate was evaluated according to the AFNOR standard NF V 05-104 [27], in which test samples are incinerated at 500 °C until a whitish powder was obtained. The pH and the titratable acidity expressed as a percentage of lactic acid were measured on a suspension made of 10 g of leaf powder in 90 mL of distilled water according to the method described by Nout et al. [28].

#### 3.3.2. Phytochemical Screening

It is a qualitative analysis based on color and/or precipitation reactions which makes it possible to establish the presence or absence of certain bioactive chemical compounds in the plant from its powder. Screening helps to look for: alkaloids, tannins (gallic and catechic), flavonoids (free flavonoids and anthocyanins), reducing compound, coumarins, saponins, sterols and tri-terpenes and cyanogenetic derivatives. These tests are carried out in the presence of certain characterization reagents according to the methods described by Harborne and Bruneton [29,30]:

1. Characterization of alkaloids

The presence of alkaloids is established by salt precipitation and revelation with Mayer’s reagent (potassium tetra-iodomercury solution).To 10 g of powder are added 50 mL of 10% H_2_SO_4_. After 24 h maceration at room temperature, the macerated material is filtered and washed with water to obtain 50 mL of filtrate. To 1mL of filtrate are added 5 drops of Mayer reagent and the mixture is left 15 min at room temperature. The presence of alkaloids is illustrated after a rapid extraction with chlorinated solvent (CHCl_3_). A sensible quantity of filtrate is made alkaline by dilution with 50% NH_4_OH and the same volume of chlorinated solvent is added. After stirring, the organic phase is removed; the remaining is filtered and then evaporated to dryness. Two milliliters of an acid solution (HCl or H_2_SO_4_) at 10% are added to the residue obtained and the mixture is poured into two test tubes. Five drops of Mayer’s reagent are added to the first tube, the second tube serves as a control. The appearance of a white-yellow or light-yellow precipitate confirms the presence of alkaloids.

2. Characterization of Tannins

The presence of gallic and catechic tannins has been demonstrated using ferric chloride. Five grams of sample powder are added to 100 mL of boiling water. After 15 min, the suspension is filtered and rinsed. This infusion will also serve to characterize the presence of flavonoids. Hydrolysable gallic tannins are evidenced by adding 15 mL of Stiasny reagent to 30 mL of the 5% infusion. After heating in a water bath at 90 °C during 15 min, the mixture is filtered and saturated with 5 g of sodium acetate, and then 1 mL of a solution of 1% FeCl_3_ is added. The appearance of a blue-black tint indicates the presence of gallic tannins. The non-hydrolyzable catechin tannins are characterized by the addition of 1 mL of conc. HCl to 5 mL of the previously prepared infusion. The mixture is boiled for 15 min. In the presence of catechin tannins, a red precipitate, insoluble in isoamyl alcohol, is formed. Catechin tannins are also evidenced after the addition of Stiasny’s reagent by the formation of a precipitate.

3. Characterization of flavonoids

The reaction with cyanidine reveals the presence of free flavonoids. To 5 mL of the former 5% infusion, are added 5mL of a mixture of ethanol and concentrated HCl (50:50, *v*/*v* %), followed by 1 mL of isoamyl alcohol and a few magnesium chips; the appearance of a pink-orange or purplish pink color reveals the presence of free flavonoids. Anthocyanins are revealed by mixing 5 mL of the infusion with either 5 mL of 10% H_2_SO_4_ or 5 mL of 50% NH_4_OH. If the color of the infusion is accentuated by acidification and then turns blue in alkaline medium, we can conclude to the presence of anthocyanins.

4. Characterization of saponins

A decoction at 1% is prepared by adding 1 g of powder to 100 mL of boiling water; a slight boiling is maintained for 15 min and then the suspension is filtered. Between 1 and 10 mL of filtrate are added successively to 10 test tubes, the volumes are made up to 10 mL with water. The contents of each tube are shaken during 15 s. The height of the resulting foam is measured 15 min after stirring. The growth index is calculated from the tube number (N) in which the foam height is 1 cm. Im = 1000/N.

5. Characterization of reducing compounds

Several reducing compounds can be detected, by preparing a 10% aqueous decoction from 10 g of plant material powder in 100 mL of water for 15 min. After evaporation to dryness of 5 mL of this 10% decoction, 3 drops of concentrated H_2_SO_4_ are added followed by 4 drops of a saturated solution of thymol in ethanol. The appearance of a red solution reveals the presence of oses and holosides. Cyanogenic glycosides are often found in plants. They are evidenced by carrying out a suspension of 1 g of plant powder in 5 mL of a solution of the same volume of water and toluene. A filter paper strip soaked with Guignard’s reagent (2 g of picric acid and 20 g of sodium carbonate in 200 mL of distilled water) is then deposited in the tube. The appearance of a red color indicates the presence of cyanogenic glycosides.

6. Characterization of sterols and triterpenes

The presence of sterols and triterpenes is demonstrated using concentrated H_2_SO_4_. An extract is first made from maceration for 24 h of 1 g of sample powder in 20 mL of ether. The extract obtained is also used for the characterization of coumarins. Sterols and triterpenes are evidenced by adding 1 mL of CHCl_3_ to the 10 mL residue of the evaporated macerate. The solution obtained is divided into two test tubes, then 1–2 mL of concentrated H_2_SO_4_ are added to the bottom of one of the tubes, the second one serves as a control. The formation of a brownish or purple red ring at the interface reveals their presence.

7. Characterization of coumarins

The presence of coumarins is achieved by evaporating to dryness 5mL of an ethereal extract. Two milliliters of hot water are added and then 1 mL of 25% NH_4_OH. The mixture is illuminated with UV light at 366 nm. An intense blue fluorescence indicates the presence of coumarins.

## 4. Conclusions

*Salvia argentea* has great potential in Algeria for the treatment of respiratory diseases. Many perspectives and expectations emerge from this study, in both the scientific and the public health domains. Thus, the continuity of this study should allow by in vitro and in vivo pharmacological approaches assessing the therapeutic efficacy attributed to *Salvia argentea* and to help clarify the cellular and subcellular mechanisms involved in the anti-inflammatory effects. Further studies would focus on the secondary metabolites and bioactive substances with the help of methods of extraction and fine characterization and should contribute to a better knowledge of the medicinal flora of the traditional Algerian pharmacopoeia.

## Figures and Tables

**Table 1:** Physicochemical parameters of the leaf powder of *Salvia argentea*.

Parameters	Powder Leaves of *Salvia argentea*
Humidity (%)	12.89 ± 1.09
Ash (%, Dry Basis)	17.61 ± 0.54
pH	8.05 ± 0.07
Titratable acidity (%)	0.74 ± 0.01

**Table 2:** Phytochemical screening results of the leaf powder of *Salvia argentea*.

Chemical Groups	Results
Alkaloids	++++
Free flavonoids	−
Anthocyanins	++++
Gallic Tannins	++++
Cathechol tannins	++++
SterolsandTerpens	+++
Coumarins	++
Saponins	++++
Oses and holosides	+++
Cyanogenetic derivatives	−

## Appendix A

1- Link: Saida, Youb, SidiBoubkeur, Hessassna, OuledBrahim, Sidi Ahmed, Moulay El Arbi, Ain El-Hadjar.
2- Informant: Age, Sex, Family situation, Academic level, Type of collector, Origin of information.
3- Use of the plant: Therapeutic, Cosmetic, Others.
4- Type of diseases treated: Dermatological, Respiratory, Cardiovascular, Genito-urinary, Osteo-articular, Metabolic, Digestive tract, Neurological, Others.
5- Used part: Leaves, Stem, Flowers, Fruits, Root, Whole plant, Others
6- Preparation: Powder, Infusion, Decoction, Poultice, Maceration, Cooked, Others.
7- Method of administration: Oral, Massage, Rinsing, Painting, Others.
8- Result: Healing, Improvement or Ineffective.
